# A protocol for CABS-dock protein–peptide docking driven by side-chain contact information

**DOI:** 10.1186/s12938-017-0363-6

**Published:** 2017-08-18

**Authors:** Mateusz Kurcinski, Maciej Blaszczyk, Maciej Pawel Ciemny, Andrzej Kolinski, Sebastian Kmiecik

**Affiliations:** 1 Faculty of Chemistry, Biological and Chemical Research Center, University of Warsaw, Żwirki i Wigury 101, 02-089 Warsaw, Poland; 2 Faculty of Physics, University of Warsaw, Pasteura 5, Warsaw, 02-093 Poland

**Keywords:** Protein–peptide interactions, Molecular docking, Flexible docking, Protein–peptide complexes

## Abstract

**Background:**

The characterization of protein–peptide interactions is a challenge for computational molecular docking. Protein–peptide docking tools face at least two major difficulties: (1) efficient sampling of large-scale conformational changes induced by binding and (2) selection of the best models from a large set of predicted structures. In this paper, we merge an efficient sampling technique with external information about side-chain contacts to sample and select the best possible models.

**Methods:**

In this paper we test a new protocol that uses information about side-chain contacts in CABS-dock protein–peptide docking. As shown in our recent studies, CABS-dock enables efficient modeling of large-scale conformational changes without knowledge about the binding site. However, the resulting set of binding sites and poses is in many cases highly diverse and difficult to score.

**Results:**

As we demonstrate here, information about a single side-chain contact can significantly improve the prediction accuracy. Importantly, the imposed constraints for side-chain contacts are quite soft. Therefore, the developed protocol does not require precise contact information and ensures large-scale peptide flexibility in the broad contact area.

**Conclusions:**

The demonstrated protocol provides the extension of the CABS-dock method that can be practically used in the structure prediction of protein–peptide complexes guided by the knowledge of the binding interface.

## Background

The prediction of protein–peptide complexes is a demanding modeling challenge, particularly when significant conformational changes occur in the binding process. The modeling of large-scale dynamics during binding cannot be effectively performed with standard simulation tools of all-atom resolution. A significant speed-up in flexible docking simulations can be achieved using coarse-grained protein models [1]. The CABS-dock is a method based on a coarse-grained model that is one of the most effective approaches to the simulations of large conformational changes during protein binding [1–3]. The CABS-dock is available as a web server [4–6]. The method doesn’t use any knowledge about peptide structure or a peptide binding site. Additional information on the protein–peptide interaction interface (obtained from experiments or theoretical predictions) may significantly improve the docking accuracy [7]. For example, the majority of state-of-the-art protein–peptide docking tools, like Rosetta FlexPepDock [8] or HADDOCK [9], follow the data-driven docking paradigm. The Rosetta FlexPepDock method enables selection of the “anchoring residue”, a residue that will be constrained during simulation on a given anchoring position. On the other hand, the HADDOCK approach uses so-called “ambiguous interaction restraints” that label receptor residues as “active” or “passive” in peptide binding.

In the CABS-dock method, the most intuitive way to introduce information about protein–peptide contact(s) is to apply distance constraint(s) on a chosen residue pair during the simulation. The side-chain contact information may be derived either directly from structural experiments or with bioinformatics tools. The possible approaches include binding site prediction [10], similarity based docking [11] or analysis of protein sequence co-evolution [12]. In this work, we present a strategy for incorporating the information on protein–peptide side-chain interactions into the CABS-dock procedure. The developed protocol for docking driven by side-chain contact information leads to a significant improvement in modeling accuracy as compared with CABS-dock docking in the default mode.

## Methods

### CABS model

The CABS-dock uses a CABS coarse-grained protein model for flexible docking simulations. The main features of the CABS model (described in detail elsewhere [13] and also in recent review [1]) are summarized below:

1. Coarse-grained representation of molecules: each amino acid residue is represented by three pseudo-atoms: **C**arbon **A**lpha (Cα), carbon **B**eta and the **S**ide-chain. To mimic the peptide bond, the fourth center of interactions is defined in the geometrical center of the virtual Cα–Cα bond. Positions of the Cα atoms are restricted to the cubic lattice, whereas other pseudoatoms are placed off the lattice.
2. Statistical force field: the energy of the complex models is related to the frequency of interactions observed in already solved structures available in the PDB [14];
3. Sampling of the configurational space is controlled by the Replica Exchange Monte Carlo scheme.

Such a design of the CABS model leads to significant simulation speed-up, by three to four orders of magnitude with regard to all-atom molecular dynamics. At the same time, reasonable resolution of modeled structures is preserved, as coarse-grained models may be easily rebuilt to realistic all-atom representation. The CABS model was successfully applied to a variety of modeling tasks including: protein structure prediction [15, 16], simulations of folding mechanisms [17–20], flexibility of globular proteins [21, 22] and modeling of protein–protein and protein–peptide complexes [23–28].

### CABS-dock docking procedure

**Fig. 1:**
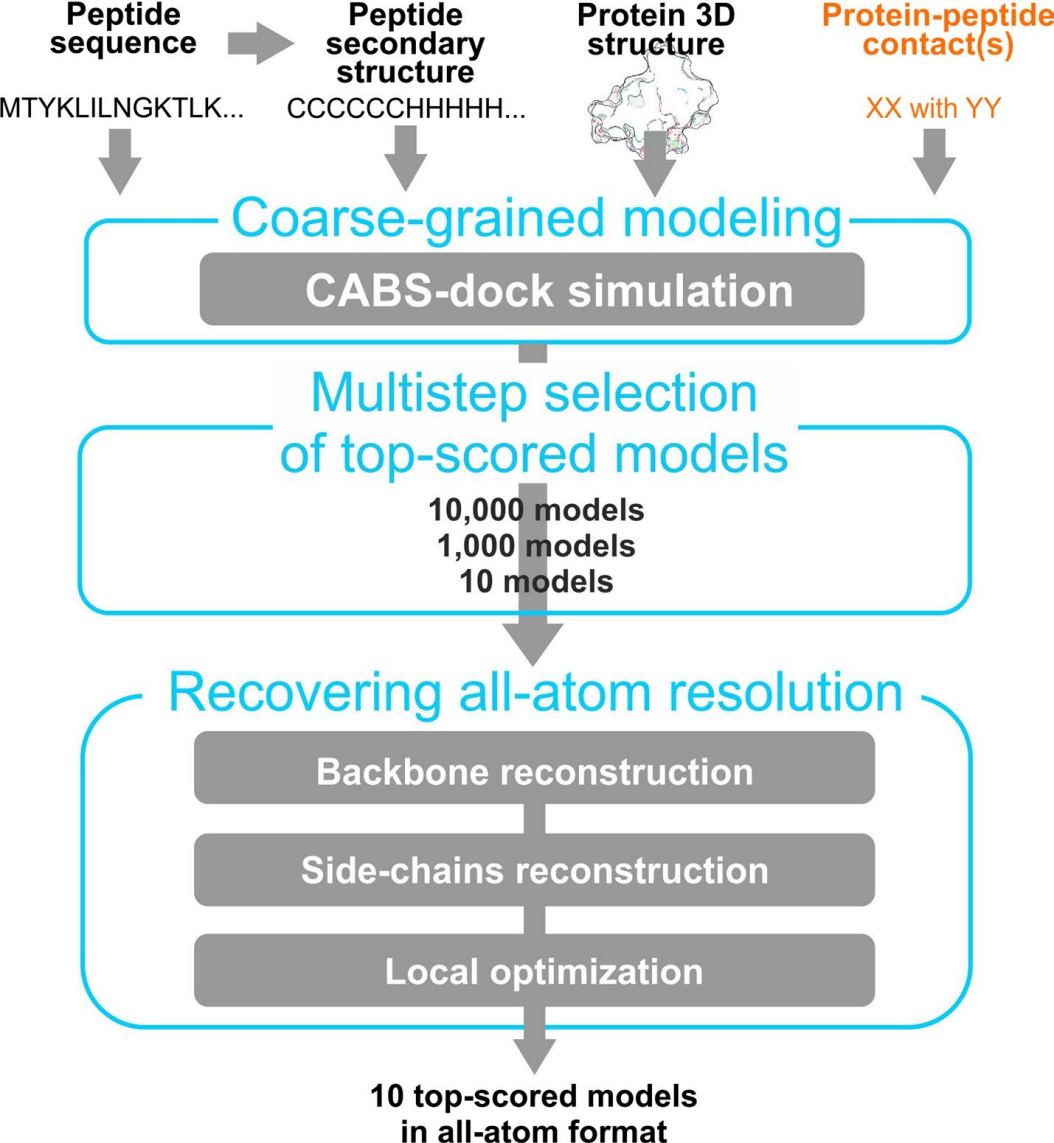
CABS-dock pipeline. The pipeline shows CABS-dock in the default mode (without any contact information) and additional input information used in the contact driven mode (marked in *orange*)

The pipeline of the CABS-dock method for protein–peptide docking [4–6] is presented in Fig. 1. The modeling procedure consists of four steps: (1) initial setup, (2) coarse-grained simulation, (3) model selection and (4) model refinement.

### Initial setup

In the initial setup the receptor structure is translated into coarse-grained representation. Subsequently, ten copies of the peptide in random conformations are generated for the replica exchange method and also transformed into coarse-grained representation. As in the default docking mode, random peptide conformations are randomly scattered around the receptor at distances up to 20 Å from the receptor molecular surface. The information about the side-chain contacts between the receptor and the peptide is transformed into soft distance restraints imposed on the modeled molecules.

### Simulation

**Fig. 2:**
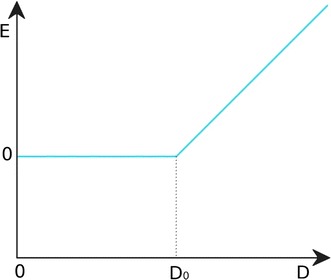
A simple attractive potential for side-chain contact. The potential introduces an energetic penalty (E) that is dependent on the distance (D) between pseudoatoms representing selected side chain contact (see also Eq. 1)

The coarse-grained simulation of the system is carried out in ten copies at different temperatures, with the exchange of coordinates between copies every given number of simulation cycles. The peptide molecule is fully flexible during the docking simulation. In the contact-driven mode of the CABS-dock method, we introduced a simple contact potential described by the following formula:

1 $$E = \left\{ {\begin{array}{*{20}c} 0 & {{\text{if }}D \le D_{0} } \\ {s(D -D_{0} )} & {{\text{if }}D > D_{0} } \\ \end{array} } \right.$$

where $D$ is the observed distance between pseudoatoms representing side chains, $D_{0}$ is the distance below which the potential vanishes and *s* is the slope of the potential line. This potential is also depicted in Fig. 2. Its role is to draw the ligand molecule to the binding site, but not to contribute to the final conformational energy of the complex. As in the default CABS-dock modeling mode [4, 5] the receptor molecule is also flexible, both on the side-chain and backbone level, but kept in near native conformation by distance restraints.

### Model selection

**Fig. 3:**
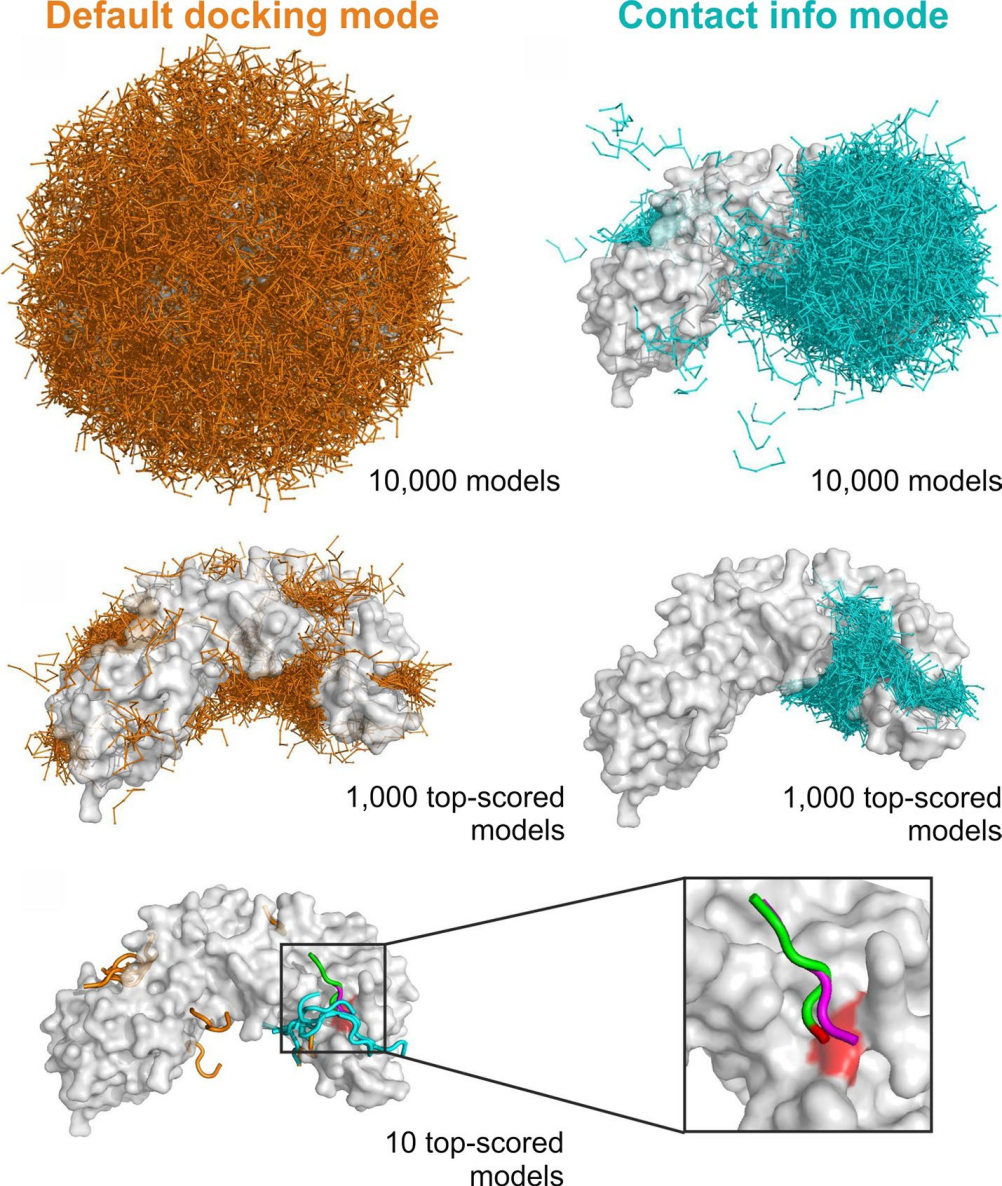
CABS-dock predictions for the 3d1e complex. The image shows CABS-dock 3d1e predictions in two docking modes: default mode (*left column*, without using any information about the binding interface) and contact information mode (*right column*, using information about a single side-chain contact). The *upper panels* show sets of 10,000 models. The *middle panels* show sets of 1000 top scored models. The *lower panel* shows sets of 10 top scored models obtained in both docking modes. Peptide models obtained in default and contact information mode are colored in *orange* and *cyan*, respectively. The peptide model with the lowest RMSD (from the contact information mode) is shown in *green*, the peptide from the experimental complex in *magenta*, and the receptor residue belonging to the side-chain contact used in the docking is marked in *red*. Ligand-RMSD between this model and the experimental peptide structure is 1.76 Å

CABS-dock simulation provides 10,000 alternative models of the complex. From this set the 1000 top scored complexes (with the lowest CABS interaction energy) are selected for the next step. Final selection is done by clustering the 1000 models using the k-medoid procedure with k = 10 and ligand RMSD (root mean square deviation of peptide coordinates after superposition of receptor molecules) as the measure of model similarity. The medoids from each cluster are selected for the next step as 10 top ranked models. The ranking from 1st to 10th is based on cluster density values (number of cluster models divided by their average difference within a cluster). Figure 3 shows consecutive stages of model selection.

### Refinement

Finally, 10 top ranked models are reconstructed to all-atom representation. For this task, CABS-dock method uses an automated Modeller procedure [29].

## Results and discussion

We tested the developed protocol (for driving CABS-dock docking with side-chain(s) contact information) on several protein–peptide complexes from previous CABS-dock tests (without contact information, default docking settings) [4, 5]. The results, together with comparison (default docking vs. docking with contact information) are presented in Table 1. In each case, a single protein–peptide contact for driving the docking was chosen randomly (see Table 2). The parameters of the attractive potential for side-chain contacts (see Eq. 1) were set as: *D*_*0*_ = 5.0 Angstroms, *s* = 1.0. Like in our previous CABS-dock tests [4, 5], preferred secondary structures of the peptides were taken from the native structures of the complexes.

**Table 1:** Comparison of CABS-dock docking performance without (default) vs. with information about a randomly selected contact

	Docking without contact information (default CABS-dock settings)				Docking driven by random contact information			
PDB	RMSD^10k^	RMSD^1k^	RMSD^100^	RMSD^10^	RMSD^10k^	RMSD^1k^	RMSD^100^	RMSD^10^
2v3s	2.42	2.42	3.48	8.89	1.30	1.37	1.65	1.77
2vj0	2.09	2.96	4.12	3.91	2.71	3.00	3.00	3.40
2zjd	2.35	2.69	2.79	4.60	1.77	2.03	2.03	3.06
3bfq	10.20	11.53	14.22	13.48	1.47	1.47	2.34	2.89
3bu3	6.87	7.06	7.71	8.86	3.62	4.45	5.33	5.47
3bwa	2.42	2.75	3.17	3.94	2.00	2.32	2.36	3.55
3cvp	4.52	4.67	8.47	10.14	2.37	2.98	3.91	4.29
3d1e	4.39	6.59	8.18	18.82	1.76	1.76	2.01	1.76
3d9t	3.56	4.34	7.25	10.06	1.96	2.69	3.42	3.72

The table shows RMSD values showing the lowest RMSD value from: 10,000 CABS-dock models (RMSD^10k^), 1000 top-scored CABS-dock models (RMSD^1k^), 100 top-scored CABS-dock models (RMSD^100^), 10 top-scored CABS-dock models (RMSD^10^)

**Table 2:** Input data for CABS-dock protein–peptide docking using information about side-chain contacts

PDB	Receptor chain	Peptide sequence	ID of contact residues	
			Receptor	Peptide
2v3s	B	GRFQVT	449	4
2vj0	A	PKGWVTFE	782	3
2zjd	A	GGDDDWTHLS	35	9
3bfq	G	ADSTITIRGYVRDNR	117	5
3bu3	A	YNPYPEDYGDIEIG	1181	11
3bwa	A	FPTKDVAL	66	1
3cvp	A	NRASKL	557	4
3d1e	A	GQLGLF	364	1
3d9t	B	ATPFQE	307	4

Single contacts were randomly selected from native contacts (defined using 5 Å distance cut-off based on positions of heavy atoms)

For most of the docking cases, we noted significant improvement (see Table 1). One of the cases (PDB ID: 3d1e) is shown in Fig. 3. For this test case, in the default CABS-dock mode (without any contact information), the accuracy of predictions in the set of ten top-scored models was very low (RMSD^10^ was 18.82 Å, the peptides are shown in orange). Side-chain contact information enables restraining the conformational sampling of a peptide to the broad neighborhood of the contact. This resulted in the selection of 10 top-scored peptides that were much closer to the binding site than in the default docking mode.

**Fig. 4:**
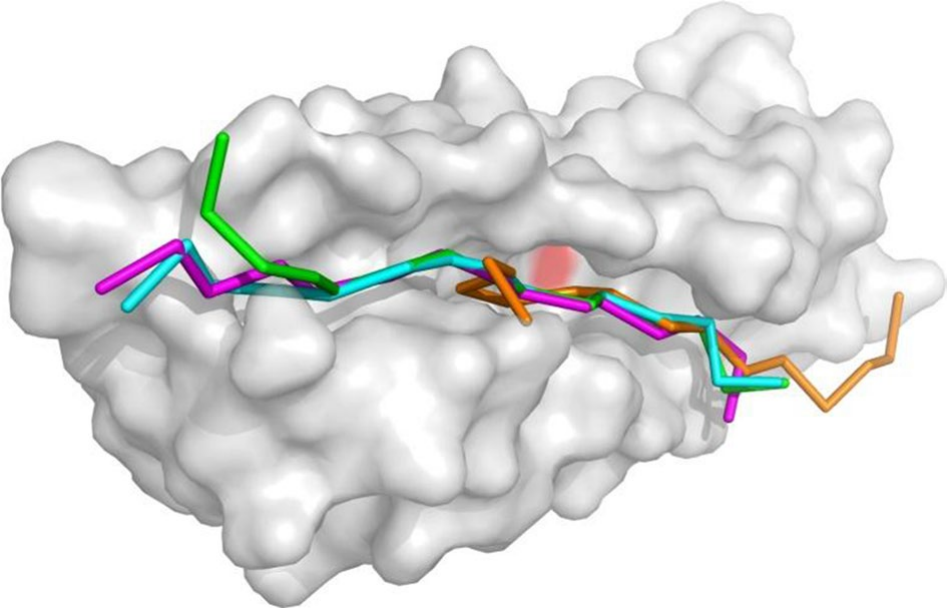
CABS-dock predictions for the 3bfq complex. The image shows comparison of the experimental peptide pose (in *magenta*, taken from the 3bfq complex) with CABS-dock models using contact information: the best from ten top-scored models (in *green*, RMSD^10^ = 2.89 Å) and the best from 10,000 models (in *cyan*, RMSD^10k^ = 1.47 Å). Additionally, the best model from 10 top-scored models without contact information (default mode) is shown (in *orange*, RMSD^10^ = 10.02 Å). The receptor residue belonging to the side-chain contact used in the docking is marked in *red*

**Fig. 5:**
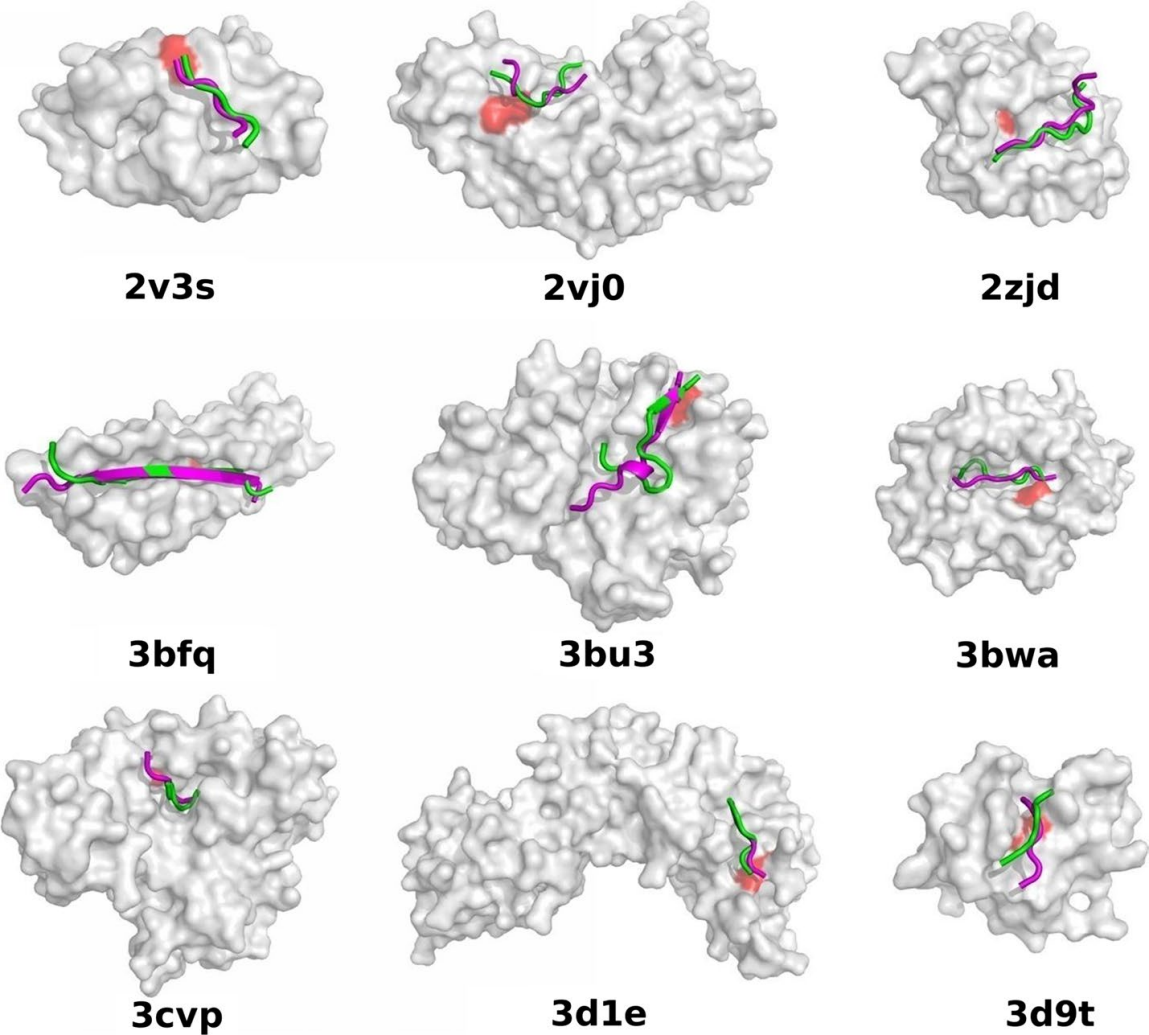
CABS-dock top-scored predictions. Peptide models with the lowest RMSD among 10 top-scored models are shown in *green*, peptides from the experimental complex in *magenta*, and the receptor residue belonging to the side-chain contact used in the docking is marked in *red*

Another docking example of 3bfq complex is presented in Fig. 4. In this case, a much longer peptide (15 residues) was docked. As compared to docking in the default mode, the use of contact information enabled significant improvement of the docking accuracy, however, there is still room for improvement. Namely, the lowest RMSD model out of the 10,000 models is much more accurate than that out of the 10 top-scored models, which is also the case for other modeled complexes (see Table 1). CABS-dock top-scored predictions for all testing cases are presented in Fig. 5.

## Conclusions

The accurate characterization of protein–peptide interfaces is important for understanding the molecular basis of life and rational design of peptide therapeutics [30]. Also, the lessons learnt from protein–peptide molecular docking can be extremely valuable in addressing important questions regarding the modeling of protein–protein interactions [31, 32].

In this work we demonstrated how very sparse and easily accessible data may improve structure prediction of protein–peptide complexes with the CABS-dock method. We introduced a simple protocol that transforms information about expected protein–peptide contacts into soft restraints, which enable extensive sampling of the peptide conformational space in a large area around the defined contact. Further development of the protocol will provide a promising tool for high-throughput studies, incorporated into a publicly available CABS-dock server. Our protocol can be easily combined with other bioinformatics tools, for contact prediction, or with experimental data [7]. The former is an especially promising approach as there already are numerous methods that could be incorporated in such a pipeline (for example binding site prediction tools [7, 33, 34]). Additional improvements can be achieved using better scoring and selection procedures that would be able to fish out the best accuracy peptide models out of a large set of CABS-dock predictions. This can be done in various ways, for example, using external force-fields (e.g. all-atom molecular dynamics [5]) or machine learning approaches [35].

## References

[1] Kmiecik S, Gront D, Kolinski M, Wieteska L, Dawid AE, Kolinski A (2016). Coarse-grained protein models and their applications. Chem Rev.

[2] Ciemny MP, Debinski A, Paczkowska M, Kolinski A, Kurcinski M, Kmiecik S (2016). Protein-peptide molecular docking with large-scale conformational changes: the p53-MDM2 interaction. Sci Rep.

[3] Antunes DA, Didier D, Kavraki LE (2015). Understanding the challenges of protein flexibility in drug design. Expert Opin Drug Discov.

[4] Kurcinski M, Jamroz M, Blaszczyk M, Kolinski A, Kmiecik S (2015). CABS-dock web server for the flexible docking of peptides to proteins without prior knowledge of the binding site. Nucleic Acids Res.

[5] Blaszczyk M, Kurcinski M, Kouza M, Wieteska L, Debinski A, Kolinski A (2016). Modeling of protein-peptide interactions using the CABS-dock web server for binding site search and flexible docking. Methods.

[6] Ciemny MP, Kurcinski M, Kozak JK, Kolinski A, Kmiecik K (2017). Highly flexible protein-peptide docking using CABS-Dock. Methods Mol Biol.

[7] Trellet M, Melquiond ASJ, Bonvin AMJJ (2015). Information-driven modeling of protein-peptide complexes. Methods Mol Biol.

[8] London N, Raveh B, Cohen E, Fathi G, Schueler-Furman O (2011). Rosetta FlexPepDock web server—high resolution modeling of peptide–protein interactions. Nucleic Acids Res.

[9] Trellet M, Melquiond ASJ, Bonvin AMJJ (2013). A unified conformational selection and induced fit approach to protein-peptide docking. PLoS ONE.

[10] Petsalaki E, Evangelia P, Alexander S, Eduardo G-U, Russell RB (2009). Accurate prediction of peptide binding sites on protein surfaces. PLoS Comput Biol.

[11] Lee H, Hasup L, Lim H, Lee MS, Chaok S (2015). GalaxyPepDock: a protein–peptide docking tool based on interaction similarity and energy optimization. Nucleic Acids Res.

[12] Hopf TA, Schärfe CPI, Rodrigues JPGLM, Green AG, Kohlbacher O, Sander C (2014). Sequence co-evolution gives 3D contacts and structures of protein complexes. Elife.

[13] Kolinski A (2004). Protein modeling and structure prediction with a reduced representation. Acta Biochim Pol.

[14] Kmiecik S, Kolinski A (2017). One-dimensional structural properties of proteins in the coarse-grained CABS model. Methods Mol Biol.

[15] Blaszczyk M, Jamroz M, Kmiecik S, Kolinski A (2013). CABS-fold: server for the de novo and consensus-based prediction of protein structure. Nucleic Acids Res.

[16] Kmiecik S, Jamroz M, Kolinski M (2014). Structure prediction of the second extracellular loop in G-protein-coupled receptors. Biophys J.

[17] Kmiecik S, Kolinski A (2007). Characterization of protein-folding pathways by reduced-space modeling. Proc Natl Acad Sci USA.

[18] Kmiecik S, Kolinski A (2008). Folding pathway of the b1 domain of protein G explored by multiscale modeling. Biophys J.

[19] Kmiecik S, Jamroz M, Kolinski A. Multiscale approach to protein folding dynamics. In: Kolinski A, editor. Multiscale Approaches to Protein Modeling. New York: Springer; 2011. p. 281–293.

[20] Jamroz M, Kolinski A, Kmiecik S (2014). Protocols for efficient simulations of long-time protein dynamics using coarse-grained CABS model. Methods Mol Biol.

[21] Jamroz M, Kolinski A, Kmiecik S (2013). CABS-flex: server for fast simulation of protein structure fluctuations. Nucleic Acids Res.

[22] Jamroz M, Kolinski A, Kmiecik S (2014). CABS-flex predictions of protein flexibility compared with NMR ensembles. Bioinformatics.

[23] Kurcinski M, Kolinski A (2007). Steps towards flexible docking: modeling of three-dimensional structures of the nuclear receptors bound with peptide ligands mimicking co-activators’ sequences. J Steroid Biochem Mol Biol.

[24] Kurcinski M, Kolinski A (2007). Hierarchical modeling of protein interactions. J Mol Model.

[25] Kurcinski M, Kolinski A (2010). Theoretical study of molecular mechanism of binding TRAP220 coactivator to Retinoid X Receptor alpha, activated by 9-cis retinoic acid. J Steroid Biochem Mol Biol.

[26] Horwacik I, Kurcinski M, Bzowska M, Kowalczyk AK, Czaplicki D, Kolinski A (2011). Analysis and optimization of interactions between peptides mimicking the GD2 ganglioside and the monoclonal antibody 14G2a. Int J Mol Med.

[27] Steczkiewicz K, Zimmermann MT, Kurcinski M, Lewis BA, Dobbs D, Kloczkowski A (2011). Human telomerase model shows the role of the TEN domain in advancing the double helix for the next polymerization step. Proc Natl Acad Sci USA.

[28] Kurcinski M, Kolinski A, Kmiecik S (2014). Mechanism of folding and binding of an intrinsically disordered protein as revealed by ab initio simulations. J Chem Theory Comput.

[29] Webb B, Sali A (2016). Comparative protein structure modeling using MODELLER. Curr Protoc Bioinform.

[30] Fosgerau K, Hoffmann T (2015). Peptide therapeutics: current status and future directions. Drug Discov Today.

[31] Verschueren E, Vanhee P, Rousseau F, Schymkowitz J, Serrano L (2013). Protein-peptide complex prediction through fragment interaction patterns. Structure.

[32] London N, Raveh B, Movshovitz-Attias D, Schueler-Furman O (2010). Can self-inhibitory peptides be derived from the interfaces of globular protein–protein interactions?. Proteins.

[33] Lavi A, Ngan CH, Movshovitz-Attias D, Bohnuud T, Yueh C, Beglov D (2013). Detection of peptide-binding sites on protein surfaces: the first step toward the modeling and targeting of peptide-mediated interactions. Proteins..

[34] Saladin A, Rey J, Thévenet P, Zacharias M, Moroy G, Tufféry P (2014). PEP-SiteFinder: a tool for the blind identification of peptide binding sites on protein surfaces. Nucleic Acids Res.

[35] Ain QU, Aleksandrova A, Roessler FD, Ballester PJ (2015). Machine-learning scoring functions to improve structure-based binding affinity prediction and virtual screening. Wiley Interdiscip Rev Comput Mol Sci..

